# Hydraulic, wash-off and sediment transport experiments in a full-scale urban drainage physical model

**DOI:** 10.1038/s41597-020-0384-z

**Published:** 2020-02-11

**Authors:** Juan Naves, Jose Anta, Joaquín Suárez, Jerónimo Puertas

**Affiliations:** Universidade da Coruña, Water and Environmental Engineering Research Team (GEAMA), Civil Engineering School, Elviña, 15071 A Coruña, Spain

**Keywords:** Hydrology, Civil engineering

## Abstract

This paper presents a dataset obtained from hydraulic and sediment transport experiments performed in a full-scale urban drainage physical model of 36 m^2^. The study seeks to accurately measure sediment mobilization through the different parts of the model (surface, gully pots and pipe system), also obtaining a precise characterization of water flow and using realistic rainfall simulator to ensure the transferability of the results. Three different rain intensities and five sediment granulometries were tested in 6 hydraulic and 23 wash-off and sediment transport experiments. The following experimental data were produced: surface elevations and 2D runoff velocities measured by visualization techniques; surface and in-pipe water depths; flow discharges, total suspended solids concentrations and particle size distribution at the entrance of the gully pots and at the pipe system outlet; and sediment mass balances. This data is optimal for developing and validating wash-off and sediment transport formulations in urban drainage models, towards better treatment and management techniques for minimizing the impact of urban surface pollutants on the environments of towns and cities.

## Abstract (summary)

**Table:** 

Measurement(s)	elevation • velocity • depth of water • fluid flow rate • suspended sediment • particle size distribution • sediment mass balance
Technology Type(s)	Structure from Motion • particle-image velocimetry • sensor • weir • filtration • laser diffraction particle size analyzer • balance
Factor Type(s)	rain intensity • sediment granulometry
Sample Characteristic - Environment	urban stormwater • urban pollution

Machine-accessible metadata file describing the reported data: 10.6084/m9.figshare.11743965

## Background & Summary

In urban environments, pollutants are accumulated during dry weather on roads, roofs and other impervious surfaces^1^. These pollutants are washed off in rain events and transported by stormwater runoff into drainage systems and eventually into aquatic media^2^, representing one of the most significant environmental issues in urban areas^3^. In this context, concentrations of total suspended solids (TSS) are typically used as indicators in the study of the transport of fine particles^4,5^, which have been found to be closely related to many pollutants, such as heavy metals and Polycyclic Aromatic Hydrocarbons (PAH)^6–8^. TSS wash-off and transport modelling is thus an important tool for the development of management and treatment techniques to minimize the environmental impact of these contaminants.

Several empirical equations and models have commonly been used in the literature to model urban wash-off^3,8–10^. However, the prediction accuracy of these lumped formulations is still quite limited^11,12^. This due to (i) difficulties in accurately measuring input variables such as the initial sediment load and characteristics, and (ii) the fact that they do not consider spatial and sediment heterogeneities. Recently, physically-based wash-off models^13–16^ have appeared as alternatives to empirical equations as a means of considering spatial heterogeneities, and to model main wash-off processes such as the detachment of particles by raindrop impacts or runoff shear, particle transport and deposition. However, this leads to an increase in the input variables needed to accurately determine and make a precise representation of the surface flow needed. Gully pot modelling^17,18^ and sewer transport modelling^19,20^, as well as interactions between surface and drainage systems^21–24^, are also key in order to provide an integrated solution to runoff TSS mobilization.

Due to the variability and randomness of the sediment build-up process^25,26^, field data uncertainties in the measurement of sediment input variables can be propagated through models and lead to unreliable results. In addition, variability and heterogeneity of rainfall and detailed geometries of urban catchments include additional uncertainties in wash-off modelling, which can lead to the incorrect evaluation of equations and models. In this regard, some authors have performed experimental studies over impervious surfaces where initial conditions are accurately determined^10,14^ in order to assess new wash-off formulations. However, the surfaces considered in these physical models are no larger than 1 m^2^, and such models do not consider gully pots interactions or typical curb flows, so their application is limited to first approximations in simple urban catchments.

In light of this, the current dataset provides a series of experiments in which variables involving sediment wash-off by rainfall and transport through gully pots and pipes were accurately measured under laboratory-controlled conditions. The experiments were performed in a full-scale street section physical model, including a 36 m^2^ rainfall simulator and two gully pots that drain runoff into a drainage pipe system. First, an accurate hydraulic characterization was performed, measuring flows and depths generated by three different rain intensities, additionally obtaining runoff velocity distributions and elevations using visualization techniques. In Naves *et al*.^27^, these data have been used to assess Large-Scale Particle Image Velocimetry (LSPIV) and Structure from Motion (SfM) techniques to accurately represent overland flow obtaining surface velocity distributions and surface elevations respectively. The videos and images provided can be of reuse as a means of optimizing visualization techniques for hydraulic modelling purposes.

Then, in a total of 23 experiments, concentrations of TSS and particle size distributions at the entrance of gully pots and at the pipe system outlet were monitored to assess wash-off and sediment transport processes from accurately measured sediment initial conditions, using the three different rainfall intensities and five sediment classes with different granulometries. The dataset is unique in that it is obtained on a 1:1 scale with realistic and very accurately measured initial conditions, and as such will allow other researchers to test models and hypotheses, thus improving our overall knowledge of urban wash-off and sediment transport. The precisely measured input variables and the TSS concentrations at gully pots have already been used in Naves *et al*.^16^ to perform a comprehensive sensitivity analysis of a recently developed physically-based wash-off model without considering uncertainties in input variables.

## Methods

### Physical model description

**Fig. 1:**
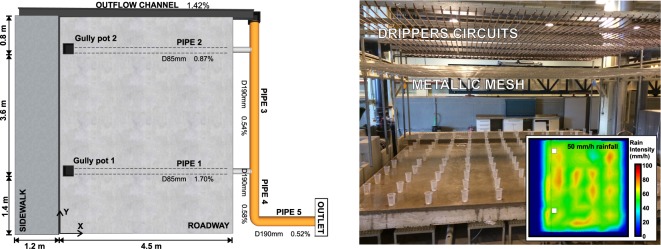
Street section physical model scheme and general image of the rainfall simulator with the experimental setup for the measurement of rain intensity maps. Results for the intermediate rainfall are also plotted.

The laboratory facility (Fig. 1a) was built in the Hydraulic Laboratory of the Centre of Technological Innovation in Construction and Civil Engineering (CITEEC) at the University of A Coruña (Spain), and consists of a rainfall simulator over a 36 m^2^ full-scale concrete street section. Two gully pots and a lateral outflow channel drain the runoff generated by rainfall into a pipe network that transports runoff to a common outlet. The rainfall simulator consists of 2,500 pressure-compensating irrigation drippers (PCJ-CNL, Netafim^TM^) disposed in a grid layout and inserted in two overlapped circuits of pipes, which are placed 2.6 m above the physical model surface. Drippers inserted in each circuit generate 1.2 and 2 L/h, respectively. Thus, given a distribution of 25 drippers per square meter in each circuit, the rainfall simulator is able to generate rainfall with a rain intensity of 30 or 50 mm/h. Both circuits can also be used at the same time generating a rain intensity of 80 mm/h. In addition, a metallic welded mesh with 3 mm square openings, located 0.6 m below the drippers, breaks and distributes drops to achieve realistic rainfall in terms of uniformity and raindrop size distribution. Rain intensity maps were measured for each rainfall from the volume collected in a 0.5 m x 0.5 m grid of vessels for 5 minutes, and are available in Naves *et al*.^28^. Figure 1b shows an image of the measuring process and the results for the intermediate rain intensity.

The street section surface model is formed by a tiled sidewalk separated by a 0.15 m high curb. Two elevation maps with different resolutions are provided, making it possible to implement the geometry in numerical models and to simulate runoff properly. First, a traditional topographic survey was carried out by measuring punctual distances from the surface to a reference laser plane with an accuracy of 0.5 mm and a resolution of 0.5 m x 0.5 m. In addition, the Structure from Motion (SfM) photogrammetric technique was used to obtain a 5 mm resolution elevation map. This technique provides a 3D reconstruction from the triangulation of different points that appear in several images of the analyzed objects. 64 raw images, the point cloud resulting from the SfM software, and the methodology and reference points used to obtain the final elevation map, are all available in Naves *et al*.^29^, and can be used to assess, analyze and reproduce the implementation of this technique to hydraulic physical models.

The generated rainfall runoff drains into the pipe system through two gully pots located along the curb and a lateral outflow channel. Then, flow is transported from the gully pots and from the end of the outflow channel to a common pipe system outlet. All the geometric details of the drainage system, such as gully pot dimensions, pipe diameters and slopes, are available in Naves *et al*.^28^ for better replicability.

### Tests procedure

Three different sets of experiments are presented: (i) hydraulic tests registering flow discharges and water depths, (ii) LSPIV experiments measuring overland velocities by this visualization technique, and (iii) wash-off and sediment transport tests measuring TSS mobilization in the different parts of the model (surface, gully-pots and pipes).

#### Hydraulic tests

**Fig. 2:**
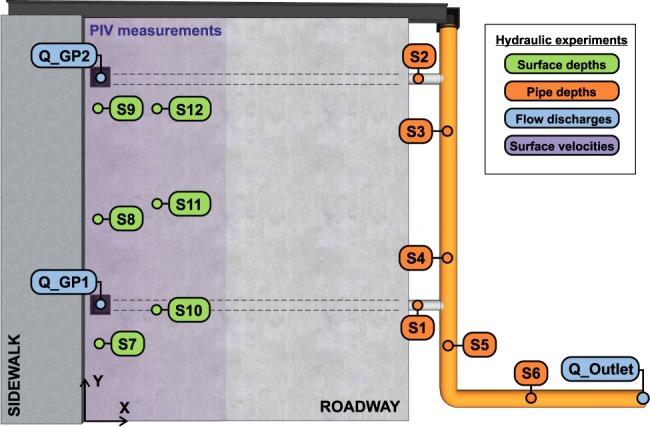
Surface and in-pipe depth, flow discharge and surface velocities measuring points in hydraulic experiments.

First, the accurate characterization of the superficial runoff and in-pipe flow play a key role in modelling wash-off and sediment transport. Therefore, the first set of experiments available consists of a detailed hydraulic characterization. In this regard, online measurements of surface and in-pipe depths and water flows in gully pots and at the pipe system outlet were registered for the three different rain intensities that the rainfall simulator was able to generate. Measuring points and sensor names are shown in Fig. 2. Further details of location, sensors used, acquisition time and units for each result are also available in the dataset^28^.

Experiments consist of simulating a steady and homogeneous rainfall of 30, 50 or 80 mm/h intensity with a duration of 5 minutes. Online measurements of surface and in-pipe depths and gully pots and pipe system outlet discharges were registered from the beginning of the rain until 5 minutes after the rain stopped. The measurement of discharge from gully pots requires closing their connection with the pipe system and deriving the gully pot inflow towards the underground deposit. This configuration prevented us from measuring total drained flow at the pipe system outlet, since the water that reached the outlet came uniquely from the outflow channel. Therefore, experiments for each rainfall were repeated with and without the connection between gully pots and the pipe system, in order to obtain discharges in the outlet and in both gully pots, respectively. Table 1 shows the six tests performed and their configuration.

**Table 1:** Hydraulic tests configurations.

Test ID	Rain intensity (mm/h)	Gully pots-pipe system configuration
HY01_30_GP	30	Disconnected
HY02_30_O	30	Connected
HY03_50_GP	50	Disconnected
HY04_50_O	50	Connected
HY05_80_GP	80	Disconnected
HY06_80_O	80	Connected

#### Large scale particle image velocimetry experiments

Six more experiments (Table 2) were performed for the LSPIV analysis in order to record surface runoff with and without fluorescent particles for each rain intensity. These experiments started by spreading particles over the model surface. Cameras began recording and ambient lights were turned off. Then, UV torches were turned on and a steady and uniform rainfall was generated. As the particles were washed-off by surface flow, it was necessary to add more particles on the roadway side during video recording. Steady runoff conditions were reached after approximately 150 seconds. For videos without particles, the same methodology was followed, but replacing the UV torches with conventional LED lamps. The first part of the test ‘Particles80’ was not recorded, and an initial recording to extract a calibration frame and a steady flow conditions video has been provided instead. Depths and discharges were not registered in this case, since they were accurately obtained in previous experiments for the same rain conditions.

**Table 2:** Configuration of LSPIV experiments.

Test ID	Rain intensity (mm/h)	Tracer	Illumination
Particles30	30	Fluorescent particles	UV
Particles50	50		
Particles80	80		
NoParticles30	30	Wave water reflections and air bubbles	LED lamps
NoParticles50	50		
NoParticles80	80		

#### Wash-off and sediment transport tests

**Fig. 3:**
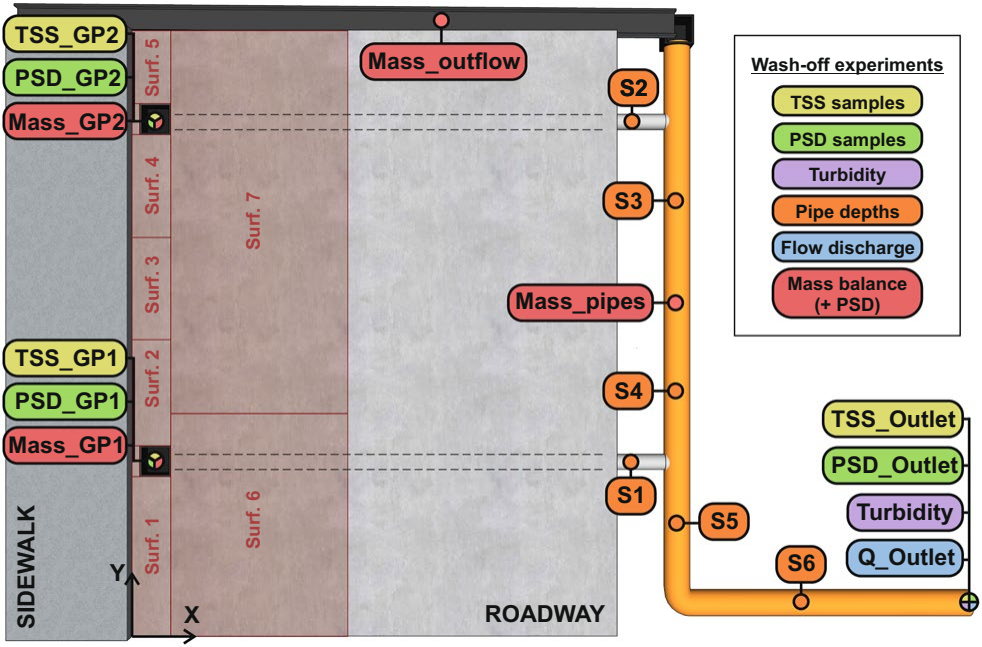
Measuring points in wash-off and sediment transport experiments.

Once the hydraulics of the experiments was accurately determined, we focused on measuring wash-off and sediment transport processes from given accurately-known initial conditions. For this, TSS and Particle Size Distribution (PSD) samples at the entrance of the gully pots and at the pipe system outlet were collected for the three rain intensities and for five sediment classes, which have different granulometries, disposed realistically over the model surface. In addition, online turbidity records were registered at the pipe system outlet, and pipe depths and outlet discharge were measured. At the end of each experiment, a mass balance was performed in order to assess the final distribution of sediment in the different parts of the physical model and to confirm the reliability of the experiments. Figure 3 shows measuring points and the identification names used.

Each test configuration combines a sediment class (D1–D5) and a homogeneous and steady rainfall (30, 50 or 80 mm/h) with a duration of 5 minutes. During the simulated event, manual grab samples in the three measurement points were collected and turbidity, pipe depths, and outlet discharge were registered up to 5 minutes after the rain stopped. Grab manual TSS and PSD samples at the entrance of the gully pots can interfere in the mass balance and in the TSS and PSD results at the pipe system outlet. Therefore, each experiment was repeated without manual samples at the gully pots, this to ensure reliability in pipe system outlet samples and final mass balances. However, some experiments with low rain intensity and larger sediment grain sizes were performed only once, since most of the sediment remained on surface or was deposited in gully pots, presenting negligible TSS concentrations at the pipe system outlet. Table 3 shows all the tests and their configurations, including those seven configurations not performed.

**Table 3:** Wash-off and sediment transport tests ID and configurations.

30 mm h^−1^ rain intensity			50 mm h^−1^ rain intensity			80 mm h^−1^ rain intensity		
Test ID	Sediment class	Measuring Point	Test ID	Sediment class	Measuring Point	Test ID	Sediment class	Measuring Point
ST01_30_D1_GP	D1	Gully pots	ST11_50_D1_GP	D1	Gully pots	ST21_80_D1_GP	D1	Gully pots
ST02_30_D1_O	D1	Outlet	ST12_50_D1_O	D1	Outlet	ST22_80_D1_O	D1	Outlet
ST03_30_D2_GP	D2	Gully pots	ST13_50_D2_GP	D2	Gully pots	ST23_80_D2_GP	D2	Gully pots
ST04_30_D2_O	D2	Not performed	ST14_50_D2_O	D2	Not performed	ST24_80_D2_O	D2	Outlet
ST05_30_D3_GP	D3	Gully pots	ST15_50_D3_GP	D3	Gully pots	ST25_80_D3_GP	D3	Gully pots
ST06_30_D3_O	D3	Not performed	ST16_50_D3_O	D3	Outlet	ST26_80_D3_O	D3	Outlet
ST07_30_D4_GP	D4	Gully pots	ST17_50_D4_GP	D4	Gully pots	ST27_80_D4_GP	D4	Gully pots
ST08_30_D4_O	D4	Not performed	ST18_50_D4_O	D4	Not performed	ST28_80_D4_O	D4	Not performed
ST09_30_D5_GP	D5	Gully pots	ST19_50_D5_GP	D5	Gully pots	ST29_80_D5_GP	D5	Gully pots
ST10_30_D5_O	D5	Outlet	ST20_50_D5_O	D5	Outlet	ST30_80_D5_O	D5	Outlet

**Fig. 4:**
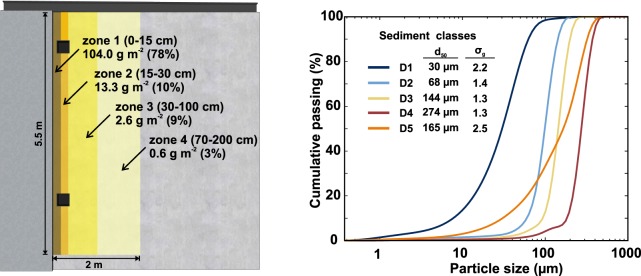
Sediment initial conditions for the wash-off and sediment transport tests. The figure includes a scheme of the stepped distribution of sediment over the model surface (**a**) and the particle size distribution of the five sediment classes used (**b**). Mean diameter and gradation coefficients (σ_g_ = $\sqrt{{D}_{84}{{/}D}_{16}}$) are also indicated in the plot.

Due to the variability and randomness of build-up process^25^, a wide range of sediment loads were measured over the urban catchments considered in the existing literature^30–33^. In this study, a load of 20 g per meter of curb was fixed as initial condition for the experiments carried out. This amount of sediment was disposed in a stepped distribution (Fig. 4a) along 5.5 m of curb following the results of Sartor and Boyd^8^, since it is known that sediments tend to be accumulated close to the curbs^34,35^. Considering that no sediment was distributed in the area of the gully pots, and that the gully pot and pipes were initially clean, the total initial mass used in each experiment was 99.44 g. The sediment used came from a roadway surface described in^36^, and was sieved obtaining four uniform granulometries (classes D1–D4 in Fig. 4b) in order to analyze the effect of sediment grain size in the wash-off and sediment transport processes. A final sediment with a continuous granulometry (class D5 in Fig. 4b), formed from a combination of the previous granulometries, was used to study the effect of employing a more realistic multi-class sediment distribution. All classes of sediment granulometries, obtained accurately by a laser coulter particle size analyzer (Beckam-Coulter LS I3 320), are available in Naves *et al*.^28^. A pycnometer was used to measure the density of the material for each sediment class, resulting in a common value of 2557 ± 16 kg/m^3^.

### Surface and pipe depths

**Fig. 5:**
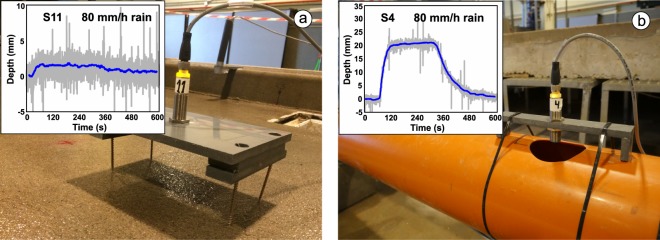
Distance sensors installed over the physical model to measure water depths. Data registered for the highest rain intensity and probes S11 (**a**) and S4 (**b**) are also shown.

A total of 6 pipe depths (S1-S6) and 6 surface depths (S7-S12) were measured using ultrasonic distance sensors (UB500-18GM75-I-V15, Pepperl and Fuchs) with a sampling frequency of 5 Hz. Prior to the experiments, sensors were pre-calibrated in order to convert the registered voltages to depths and discharges. To do this, signal-distance linear calibrations, with a determination coefficient (R^2^) above 0.99, were obtained measuring five known distances from sensors to a reference plane. After this, the raw time series registered in the experiments were processed in the following way. First, a five seconds wide median filter was implemented to remove peak signals higher than twice the standard deviation. Then, a 20 seconds wide moving median was applied to smooth the signal. Finally, the pre-calibration was used to transform signals into distances and obtain depth results from the differences between the measurements during the experiment and the dry surface reference, which was measured for 60 seconds before the rain began. Figure 5 includes images of sensors installed on the street surface (left) and pipes (right), and some examples of depth results. Calibration data, raw signals, and processed results are available in Naves *et al*.^28^ for other authors to use.

### Flow discharge

**Fig. 6:**
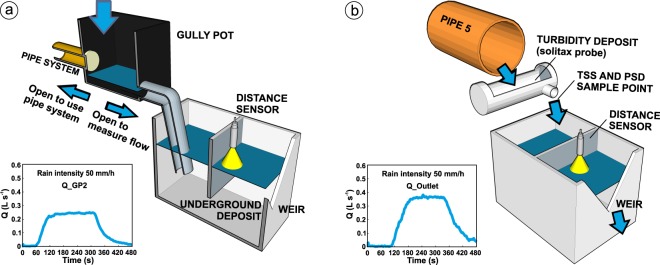
Setups for the measurement of flow discharges from the water level over a v-notch. The flow discharges registered at gully pots (**a**) and at the pipe system outlet (**b**) for the rain intensity of 50 mm/h are also plotted.

Discharges in gully pots and at the pipe system outlet were measured using ultrasonic distance sensors (UB500-18GM75-I-V15, Pepperl and Fuchs) registering the level over a v-notch weir in three different deposits. The measurement frequency was set to 2 Hz. The first deposit was 0.5 m × 0.6 m size and was installed at the pipe system outlet. The pipe outflow, after passing through a 0.4 m length and 0.16 m diameter deposit used in wash-off experiments, flowed into the deposit to obtain continuous measurements. Two additional deposits were installed bellow the gully pot in order to derive inflows as a way of measuring discharges following the same methodology. Figure 6 show schemes of the configurations of the deposits and includes examples of the processed data available in^28^.

**Fig. 7:**
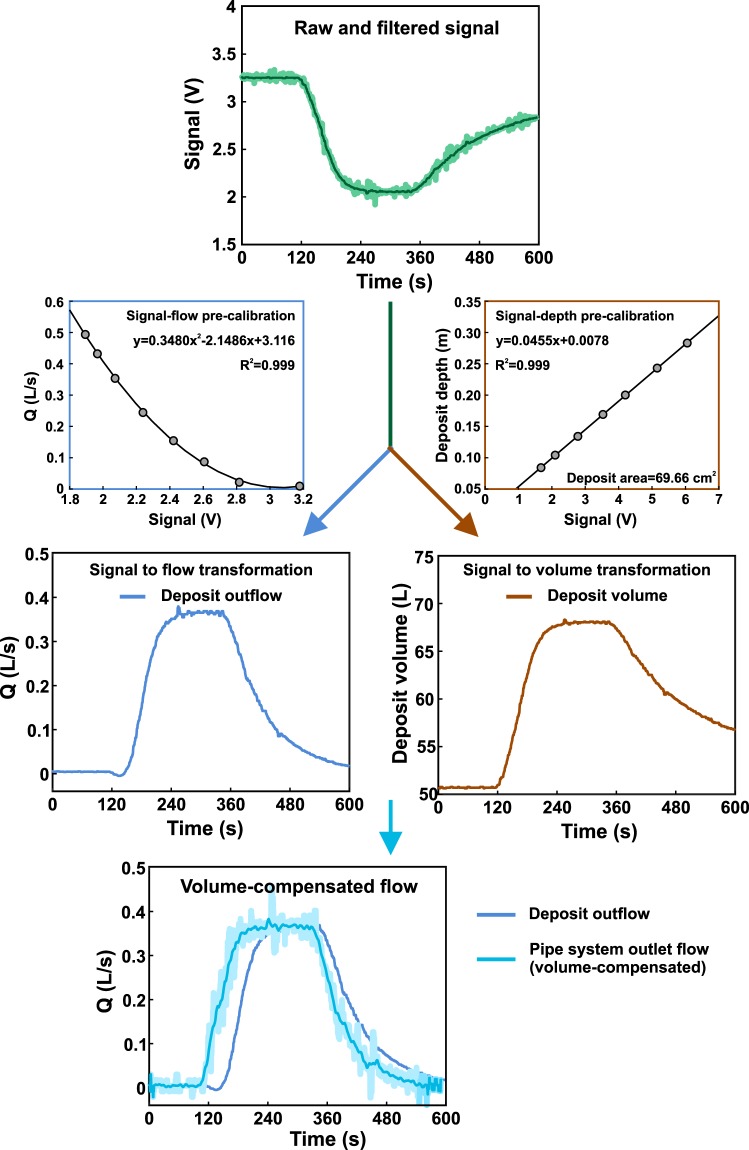
Flow discharge data processing from the raw recorded signal to the flow result at the pipe system outlet for the intermediate rain intensity.

A signal-flow pre-calibration was performed for each sensor installed in these deposits. To do this, different known steady flows were introduced in the deposit to obtain a second-degree polynomial relation (obtaining R^2^ over 0.99) in order to transform the signal recorded into discharge. The signal processing starts by removing peaks higher than twice the standard deviation with a five seconds-wide median filter. Then, a five seconds-wide moving median was applied to the signal before obtaining flow time series using pre-calibration polynomial regression. However, these results corresponded to the deposit outflow, so it was necessary to consider the volume that was retained when the flow, and thus the water level in the deposits, varied. Signal-depth calibrations, as performed for surface and pipe depths, plus the area of the deposits, were thus used to consider these variations of volume in time steps of 5 seconds, obtaining the water flow at the entrance of the deposits. Finally, flow time series were smoothed by a 20 seconds-wide moving median. Figure 7 shows the data processing procedure for the flow at the pipe system outlet generated by the intermediate rain intensity. Data regarding pre-calibrations and raw and processed signals are provided for each hydraulic experiment in Naves *et al*.^28^.

### Surface velocities

Surface flow velocities were measured using the LSPIV technique, which analyses the displacement between two consecutive frames of tracers that follow the studied flow. Frames were obtained from 4 k and 25 fps videos recorded by two Lumix GH4 cameras (focal length 28 mm), which were installed above the sidewalk of the physical model, covering the first two meters of roadway from the curb. Two different tracers were used in the experiments applying LSPIV: i) fluorescent particles illuminated by UV torches, and ii) water reflections and small bubbles generated by raindrop impacts. The analysis of the runoff velocities in steady conditions started by extracting 1,500 frames (60 s) from each camera recording. First, a spatial calibration was required to rectify the angle of the cameras and the lens distortion, and to join the images from each camera. Matlab algorithm ‘fitgeotrans’ was applied, identifying in each video reference points drawn in the model surface in order to transform video frames to an orthogonal reference system. Then, frames were converted to greyscale and a sliding background filter was applied, transforming to black those pixels with 25% or less relative difference in the grey value with the same pixel from the previous frame. The LSPIV tool for Matlab PIVLab^37^ was used to obtain raw velocities from each pair of consecutive frames (using interrogation areas of 128, 64 and 32 pixels as settings). Finally, after a temporal 2D filter^38^ and a spatial median filter of 3 × 3 elements using the Matlab toolbox ‘pivmat’, the surface velocity distribution was obtained from the mean of all the results. For the videos recording water reflections and bubbles without particles, the same methodology was followed, but using a sliding background threshold of 10%.

**Fig. 8:**
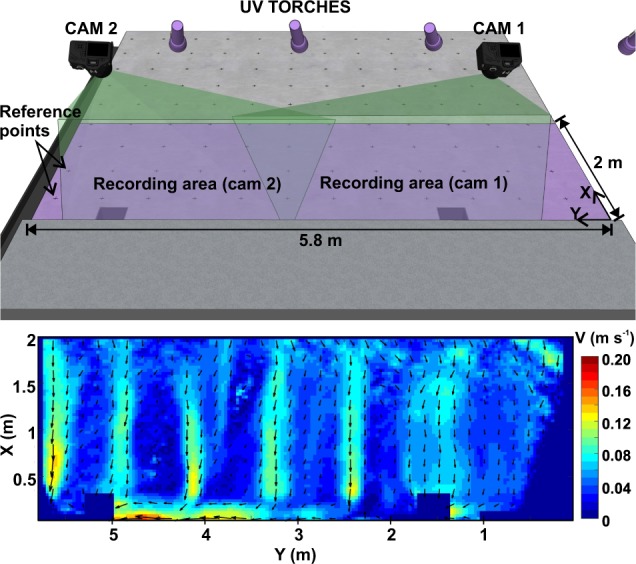
Experimental setup scheme for the recording of runoff videos used in the LSPIV analysis with fluorescent particles. The resulted steady velocity distribution generated by the lowest rainfall intensity is also plotted.

Figure 8 shows a scheme of the experimental setup and the steady results for the rain intensity of 30 mm/h obtained using fluorescent particles as tracers. Raw videos, frames processed for the LSPIV analysis, and velocity distribution results are all provided in Naves *et al*.^39^ for the use of other researchers. Further details of the fluorescent particles, the coordinates of reference point used, and the frames extracted from videos for the analysis can be also consulted.

### Total suspended solids measurements

**Fig. 9:**
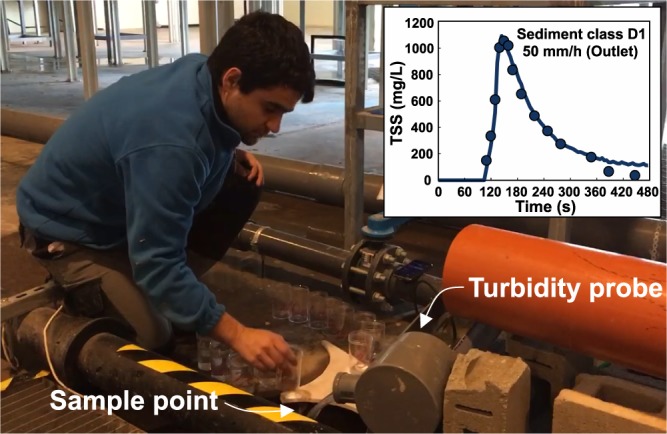
TSS results at the pipe system outlet. The plot includes TSS samples results and on-line TSS record obtained from the turbidity probe for the test ST12. Informed consent was obtained from the participant prior to publication of the image.

For each measuring point and experiment, the dataset^28^ provides the times at which manual samples were collected and the TSS concentration resulting from filtering of the samples following the^40^ standard methods. Samples were roughly 180 ml volume and were collected once a perimeter channel and a small deposit concentrated the flow at the entrance of the gully pots and at the pipe system outlet, respectively. The sample times were different for each rain intensity, since samples were taken from the moment that flow reached the measuring points until the physical model drained the water that remained after the rain stopped. In addition, the time between samples was shorter at the beginning of the experiment, this in order to detect the TSS peak. Results also include online TSS measurements at the pipe system outlet, which were obtained from the linear calibration of the signal of a turbidity probe (Solitax sc, Hach) using the manual grab samples from each experiment (Fig. 9).

### Particle size distributions

Additional manual grab samples collected at the entrance of the gully pots and at the pipe system outlet were also analyzed with a laser coulter particle size analyzer to determine grain sizes that were being washed at each sample time. In the experiments with realistic and continuous granulometry (sediment class D5), a detailed characterization of the temporal variations of the PSD in gully pots and pipe system outlet were performed with a mean of eight PSD samples for each measuring point. The number of samples was lower for experiments with uniform granulometries (sediment classes D1–D4) since no significant temporal variations of the PSD were expected. PSD raw data and sample times are included in Naves *et al*.^28^ for each measuring point.

### Mass balances

The masses of sediment collected at the end of each experiment from surface, gully pots, and pipe system are also available in the dataset^28^. This makes it possible to perform mass balances following the methodology used in Naves *et al*.^41^ in order to analyze where the sediment is deposited depending on rain intensity and on sediment grain size. It may also be an useful indicator of the reliability of the results, in that it shows how much mass was controlled. Firstly, sediment masses that remained over the surface and inside gully pots were collected with an industrial vacuum with a 98% sweeping efficiency. As can be seen in Fig. 3, the surface was divided into 7 areas in order to analyze the final distribution of sediment over the street surface. Finally, pipes were cleaned using a pressure washer, and sediment deposited in pipes was collected with a 10 µm sieve at the pipe system outlet. To close mass balances adequately, it was necessary to take into account concrete particles, which were eroded from the model surface during the vacuuming process. To do this, eight blank experiments without sediments were performed following the same procedure to determine a mean concrete mass to subtract from the masses collected in the experiments. In this way, it was possible to consider exclusively the sediment mass in the mass balances. Blank masses collected are also available in Naves *et al*.^28^. The PSD of mass balance samples were also measured using a laser coulter particle size analyzer (Beckam-Coulter LS I3 320) in order to analyze the deposition of the different grain sizes at the end of the experiments.

## Data Records

Three data packages are available in open access from the Zenodo data repository within the scope of the WASHTREET project, at the website https://zenodo.org/communities/washtreet. A main data package^28^ contains the data related with the hydraulic, wash-off, and sediment transport experiments described in this paper. Two additional packages^29,39^ are provided to include detailed raw and processed data regarding the LSPIV analysis and the SfM photogrammetric technique, respectively. A description of each data package is included below.

### Hydraulic, wash-off and sediment transport experiments data

This main data package^28^ includes further details of the physical model, rain intensity maps, surface topographies, an extended description of the materials and methods used, and raw and processed data of hydraulic (except surface velocities data) and wash-off and sediment transport experiments. In addition, it provides a series of images and videos for a better understanding of the data. Data is provided in ‘csv’ files and is organized following the tests ID showed in Tables 1 and 3 and the measuring points labels showed in Figs. 2 and 3.

### Large scale particle image velocimetry analysis data

The LSPIV data package^39^ provides, first, a detailed description of the methodology, data processing, and results. Raw 4 K videos recorded with and without particles are also available for the three rain intensities. It also provides coordinates, calibration frames, and instructions to rectify video frames to an orthogonal reference system, plus the processed frames to perform the LSPIV analysis. Finally, the steady velocity distributions obtained from each video analysis are provided.

### Structure from motion topography data

The 64 raw images used to apply the photogrammetric technique to the model surface are provided in this data package^29^. In addition, a detailed descriptions of the methodology, the raw point cloud obtained from the SfM software, and the elevation map resulted, are also available in this data package^29^.

## Technical Validation

### Hydraulic variables

Water depths and flow discharges were measured using ultrasound probes. As explained in methods section, individual calibrations were performed for each ultrasound probe by regression polynomials to transform registered voltage to water depths and flows, obtaining determination coefficients (R^2^) above 0.99 in all the cases. In Fig. 7, it can be seen examples of the good fit of the regressions performed for the ultrasound probe installed at the pipe system outlet. The uncertainties related with the ultrasound probes and the water meter used in calibration were also assessed in previous tests to check the reliability of the results obtained. First, the uncertainty in the signal registered by the ultrasound probes, which have a maximum error of 1% and a resolution of 0.13 mm, was analyzed by measuring seven different steady water levels during 1 minute and with a measurement frequency of 2 Hz. Table 4 includes the signal standard deviation for each water level, which takes values around 0.04 V and is independent of the measured valued. Then, the water discharge uncertainty in the determination of the v-notch weirs rating curves was also assessed by measuring thirteen repetitions of five different steady discharges, where the total volume was measured using a flow meter, which have a maximum error of 2%, for a duration of 3 minutes. Table 5 indicates that the standard deviations resulted are between two and three orders of magnitude lower that the measured flow discharges. Finally, distances for the signal-depth calibration were measured with a maximum error of 0.5 mm. Raw data and calibration measurements were included in the data set provided^28^ for others to use and check the reliability of the results.

**Table 4:** Standard and maximum deviation of ultrasound probe signals measuring 60 seconds at 2 Hz for seven different constant distances.

	Mean value (V)	Standard deviation (V)	Maximum deviation (V)
V1	1.681	0.04	0.211
V2	2.104	0.04	0.175
V3	2.783	0.038	0.175
V4	3.534	0.04	0.204
V5	4.203	0.039	0.17
V6	5.156	0.044	0.22
V7	6.056	0.035	0.167

**Table 5:** Standard and maximum deviation of thirteen repetitions of water flow measurements using the flow meter for five different discharges.

	Mean value (L/s)	Standard deviation (L/s)	Maximum deviation (L/s)
Q1	0.0387	0.0004	0.0008
Q2	0.155	0.0005	0.0009
Q3	0.2595	0.0004	0.0007
Q4	0.4567	0.0012	0.0019
Q5	0.6578	0.0016	0.0037

As seen in Table 1, hydraulic experiments were repeated for each rain intensity with the gully pots disconnected and connected to the pipe system. These configurations allow measuring both the flow discharge at the gully pots, measuring also outflow channel discharge at the outlet, and the total drained water flow at the pipe system outlet, respectively. Therefore, the reliability of measured flow discharges can be also assessed by comparing the total water flow registered in steady conditions at the pipe system outlet in hydraulic tests HY02, HY04 and HY06, and the sum (Q_total) of the steady water discharges measured at both gully pots and at the pipe system outlet (which correspond with the flow drained by the outflow channel, see Fig. 1) in tests HY01, HY03 and HY05, respectively. Table 6 includes the steady water flows registered in the experiments and the error obtained performing this comparison. The differences below 1.6% showed a very good agreement of the results.

**Table 6:** Comparison between the sum (Q_total) of the steady water discharges drained by gully pots and by the outflow channel measured with the gully pots disconnected to the pipe system, and steady water flow registered at pipe system outlet with the gully pots connected.

Test	Q_GP1 (L/s)	Q_GP2 (L/s)	Q_outflow channel (L/s)	Q_total (L/s)	Test	Q_outlet (L/s)	Error (%)
HY01_30_GP	0.0472	0.1447	0.0156	0.2075	HY02_30_O	0.2082	0.3
HY03_50_GP	0.0888	0.244	0.0336	0.3664	HY04_50_O	0.3661	−0.1
HY05_80_GP	0.1414	0.3402	0.0578	0.5394	HY06_80_O	0.5311	−1.6

### Surface velocities

In the determination of overland flow velocities, the following sources of uncertainty in the LSPIV procedure have been considered: (i) calibration of cameras and rectification of frames, (ii) application of sliding background filter to reduce the effect of background image features, (iii) filtering of velocity results, and (iv) depth-average velocities estimation. First, maximum errors of around 2% in the distances between reference points in rectified images were found in the calibration process, which was repeated for each test to avoid that small movements of the camera introduced additional uncertainties. Then, the sliding background was used to prevent the influence of the background image features, such as temporarily settled particles or surface roughness, in the estimation of surface velocities. The velocity of these features would be zero and the mean velocity in the interrogation areas would be reduced. The implemented 2D filter detected less than the 8% of the velocity vectors for the three rain intensities considered, indicating a good stability of the LSPIV results. Finally, the resulted velocity distributions that are provided correspond to the surface flow velocity and not to the depth-average velocities. Some authors use a flow velocity correction factor from 0.6 to 1 based on the log-law velocity profile^24,42^, and in Naves *et al*.^27^ the classical value of 0.85 was applied. However, due to the very shallow flow conditions, this assumption is not expected to add significant uncertainties.

In addition, the raw and processed data provided were used in Naves *et al*.^27^ to assess LSPIV and SfM techniques, showing their usefulness for hydraulic modelling purposes. In this work, the topographic data obtained from the photogrammetric technique was used to represent overland flow with a 2D shallow water model. The model was calibrated using water discharges at the gully pots and the surface velocity distributions obtained were compared with experimental results from the LSPIV technique. The good fit between velocity distributions showed LSPIV technique as an optimal tool to accurately measure overland flows in very shallow flows and with the presence of raindrops.

### Sediment transport experimental data

The variability involved in sediment transport processes makes the validation of the experimental data needed to check the capability of measuring TSS mobilization through the different parts of the model. As explained in methods section, a sediment mass balance was performed at the end of each experiment to assess the final distribution of sediment in the different parts of the physical model, being an optimal tool to ensure the reliability of the experimental results in that it shows how much mass was controlled. The maximum errors in the mass balances between the initial surface sediment mass and the masses collected at the end of the experiments plus the sediment washed from the physical model during the experiments were below 8% with a RMS of roughly 4%. These results are very satisfactory in consideration of the physical phenomena under study and quantify the accuracy of the experimental results provided.

In addition, nine different combinations of three TSS concentrations (300, 600 and 900 mg/L) and five sediment grain sizes (63, 250, 400, 630 and 1000 µm) were chosen to analyze the uncertainty of TSS sample determination. Thirteen replicates were filtered for each case and the mean value and the deviations resulted are shown in Table 7. It can be seen that mean errors are roughly below 2% and maximum standard deviations resulted in values bellow 7.5 mg/L, so the reliability of the TSS results obtained from manual samples was demonstrated. The possible sources of error in the online TSS records, obtained by performing a regression between TSS samples and turbidity measurements at the pipe system outlet, were also analyzed. The variability of the turbidity probe was assessed by measuring 300 s at 0.2 Hz for six different constant turbidity values. The results in Table 8 showed maximum standard deviation of 4.1% from the mean value and suitable maximum deviations to precise determine outflow turbidity. Regarding the polynomial regression between turbidity and TSS measurements performed for each test, mean determination coefficient (R^2^) of 0.94 indicated a good correlation, with the exception of experiments using coarsest sediment particles and lower rain intensities where maximum TSS concentrations are below 20 mg/L. Finally, the laser coulter particle size analyzer (Beckam-Coulter LS I3 320), which is used to analyze particle size distributions, is able to measured particle size volumetric distributions between 0.017 and 2000 µm with an error of 1% about mean size.

**Table 7:** TSS replicates results for different concentrations and sediment grain sizes. The mean value, the mean error from the reference value, and the standard and maximum deviation of 13 replicates for each case were included.

Grain size (μm)	TSS (mg/L)	Mean value (mg/L)	Mean error (%)	Standard deviation (mg/L)	Maximum deviation (mg/L)
63	300	294.7	1.75	7.4	18.5
63	600	596.1	0.64	7.3	13.5
63	900	898.5	0.17	4.1	10.2
250	300	302.4	0.79	4.5	10.1
400	300	302.2	0.72	3.8	9.1
400	600	604	0.66	3.3	8.8
400	900	904.7	0.52	4.1	9.5
630	300	301.6	0.55	4.3	7.6
1000	300	304.5	1.49	3.6	7.7

**Table 8:** Standard and maximum deviation of turbidity probe signals measuring 300 seconds at 0.2 Hz for six different constant turbidity values.

	Mean value (NTU)	Standard deviation (NTU)	Maximum deviation (NTU)
Turb1	7.5	0.1	0.3
Turb2	27.1	1.1	4.9
Turb3	58.9	1.4	4.7
Turb4	105.2	3.3	8.4
Turb5	138	5.1	18.9
Turb6	173.9	5.6	13.6

## Data Usage

Given the lack of data for the development of empirical and physically based urban wash-off models, plus the difficulty in conducting field studies accurately measuring key input variables such as initial load of sediment, its spatial distribution, or the sediment characteristics, we have proposed here a series of experiments in which the different processes are accurately measured in laboratory-controlled conditions. Our experiments are unique in that they are performed in a full-scale physical model using a very realistic rainfall simulator. In addition, not only are the initial sediment conditions completely defined, but the hydraulic behavior of the experiments is also accurately determined, including depth and velocity measurements of very shallow flows. Finally, surface wash-off and in-pipe sediment transport is precisely measured by TSS and PSD samples and by analyzing the mobilization of sediments through performing mass balances at the end of the tests. The dataset introduced in this study, then, is useful in the following ways:

- Hydraulic and wash-off dual drainage modelling studies in urban catchments. Data can be used to develop, calibrate and validate urban drainage models, including wash-off and sediment transport processes in the different components of the drainage system (surface, gully pots, in-pipe) without considering uncertainties in the input variables. The use of different sediment classes also means that the analysis of single and multi-class sediment transport modelling is itself of interest. Finally, the data herein lead to our research being replicable, and thus allows other researchers to test their own models and hypotheses.
- Assessment and optimization of seeded and unseeded LSPIV techniques. The raw videos and data provided are of great use as a means of improving the understanding of the use of LSPIV analysis in urban environments, with very shallow flows and in the presence of raindrops.
- Study of the novel application of photogrammetric techniques for hydraulic modelling purposes. The raw images and raw point cloud results can be used to assess photogrammetric techniques in order for these to be used in further field and physical model applications.

## References

[1] Zafra C, Temprano J, Suárez J (2017). A simplified method for determining potential heavy metal loads washed-off by stormwater runoff from road-deposited sediments. Sci. Total Environ..

[2] Anta J, Peña E, Suárez J, Cagiao J (2006). A BMP selection process based on the granulometry of runoff solids in a separate urban catchment. Water Sa..

[3] Egodawatta P, Thomas E, Goonetilleke A (2007). Mathematical interpretation of pollutant wash-off from urban road surfaces using simulated rainfall. Water Res..

[4] Rossi L, Chèvre N, Fankhauser R, Krejci V (2009). Probabilistic environmental risk assessment of urban wet-weather discharges: an approach developed for Switzerland. Urban Water J..

[5] Sikorska AE, Del Giudice D, Banasik K, Rieckermann J (2015). The value of streamflow data in improving TSS predictions–Bayesian multi-objective calibration. J. Hydrol..

[6] Herngren, L. F. *Build-up and Wash-off Process Kinetics of PAHs and Heavy Metals on Paved Surfaces Using Simulated Rainfall* (Doctoral dissertation, Queensland University of Technology, 2005).

[7] Akan, A. O. & Houghtalen, R. J. *Urban hydrology, hydraulics, and stormwater quality: engineering applications and computer modelling* (John Wiley & Sons, 2003).

[8] Sartor, J. D. & Boyd, G. B. *Water Pollution Aspects of Street Surface Contaminants*, EPA-R2-72-081 (United States Environmental Protection Agency, 1972).

[9] Leutnant D, Muschalla D, Uhl M (2018). Statistical distribution of TSS event loads from small urban environments. Water.

[10] Muthusamy M (2018). Improving understanding of the underlying physical process of sediment wash-off from urban road surfaces. J. Hydrol..

[11] Schellart ANA, Tait SJ, Ashley RM (2010). Towards quantification of uncertainty in predicting water quality failures in integrated catchment model studies. Water Res..

[12] Gorgoglione A (2019). Uncertainty in the parameterization of sediment build-up and wash-off processes in the simulation of sediment transport in urban areas. Environ. Modell. Softw..

[13] Deletic A, Maksimovic E, Ivetic M (1997). Modelling of storm wash-off of suspended solids from impervious surfaces. J. Hydraul. Res..

[14] Shaw SB, Walter MT, Steenhuis TS (2006). A physical model of particulate wash-off from rough impervious surfaces. J. of Hydrol..

[15] Hong M, Bonhomme C, Le MH, Chebbo G (2016). A new approach of monitoring and physically-based modelling to investigate urban wash-off process on a road catchment near Paris. Water Res..

[16] Naves J, Rieckermann J, Cea L, Puertas J, Anta J (2020). Global and local sensitivity analysis to improve the understanding of physically-based urban wash-off models from high-resolution laboratory experiments. Sci. Total Environ..

[17] Deletic A, Ashley R, Rest D (2000). Modelling input of fine granular sediment into drainage systems via gully-pots. Water Res..

[18] Post JAB (2016). Monitoring and statistical modelling of sedimentation in gully pots. Water Res..

[19] Mannina G, Schellart ANA, Tait S, Viviani G (2012). Uncertainty in sewer sediment deposit modelling: Detailed vs simplified modelling approaches. Phys. Chem. Earth.

[20] Hannouche A, Joannis C, Chebbo G (2017). Assessment of total suspended solids (TSS) event load and its uncertainties in combined sewer system from continuous turbidity measurements. Urban Water J..

[21] Djordjević S (2013). Experimental and numerical investigation of interactions between above and below ground drainage systems. Water Sci. Technol..

[22] Fraga I, Cea L, Puertas J (2015). Validation of a 1D-2D dual drainage model under unsteady part-full and surcharged sewer conditions. Urban Water J..

[23] Rubinato M (2017). Experimental calibration and validation of sewer/surface flow exchange equations in steady and unsteady flow conditions. J. Hydrol..

[24] Martins R (2018). On the Characteristics of Velocities Fields in the Vicinity of Manhole Inlet Grates During Flood Events. Water Resour. Res..

[25] Wijesiri B, Egodawatta P, McGree J, Goonetilleke A (2015). Process variability of pollutant build-up on urban road surfaces. Sci. Total Environ..

[26] Sandoval S, Vezzaro L, Bertrand-Krajewski JL (2018). Revisiting conceptual stormwater quality models by reconstructing virtual state variables. Water Sci. Technol..

[27] Naves J, Anta J, Puertas J, Regueiro-Picallo M, Suárez J (2019). Using a 2D shallow waters model to assess Large-scale Particle Image Velocimetry (LSPIV) and Structure from Motion (SfM) techniques in a street-scale urban drainage physical model. J. Hydrol..

[28] Naves J, Anta J, Suárez J, Puertas J (2019). Zenodo.

[29] Naves J, Anta J, Regueiro-Picallo M, Suárez J, Puertas J (2019). Zenodo.

[30] Zafra CA, Temprano J, Tejero I (2008). Particle size distribution of accumulated sediments on an urban road in rainy weather. Environ. Technol..

[31] Miguntanna NP, Goonetilleke A, Egodowatta P, Kokot S (2010). Understanding nutrient build-up on urban road surfaces. J. Environ. Sci..

[32] Chow MF, Yusop Z, Abustan I (2015). Relationship between sediment build-up characteristics and antecedent dry days on different urban road surfaces in Malaysia. Urban Water J..

[33] Morgan D, Johnston P, Osei K, Gill L (2017). Sediment build-up on roads and footpaths of a residential area. Urban Water J..

[34] Grottker M (1987). Runoff quality from a street with medium traffic loading. Sci. Total Environ..

[35] Deletic A, Orr DW (2005). Pollution buildup on road surfaces. J. Environ. Eng..

[36] Fraga I (2016). Global Sensitivity and GLUE-Based Uncertainty Analysis of a 2D-1D Dual Urban Drainage Model. J. Hydraul. Eng..

[37] Thielicke W. & Stamhuis E. J. PIVlab – towards user-friendly, affordable and accurate digital particle image velocimetry in MATLAB. *J. Open Res. Softw*. **2**, article e30 (2014).

[38] Goring DG, Nikora VI (2002). Despiking acoustic Doppler velocimeter data. J. Hydraul. Eng..

[39] Naves J, Anta J, Suárez J, Puertas J (2019). Zenodo.

[40] APHA. *Standard Methods for the Examination of Water and Wastewater*. (American Public Health Association, 1995).

[41] Naves J (2017). Experimental study of pollutant washoff on a full-scale street section physical model. Water Sci. Technol..

[42] Leitão JP, Peña-Haro S, Lüthi B, Scheidegger A, de Vitry MM (2018). Urban overland runoff velocity measurement with consumer-grade surveillance cameras and surface structure image velocimetry. J. Hydrol..

